# Toward Flexible and Wearable Embroidered Supercapacitors from Cobalt Phosphides-Decorated Conductive Fibers

**DOI:** 10.1007/s40820-019-0321-x

**Published:** 2019-10-17

**Authors:** Jianfeng Wen, Bingang Xu, Jinyun Zhou

**Affiliations:** Nanotechnology Center, Institute of Textiles and Clothing, The Hong Kong Polytechnic University, Hung Hom, Kowloon, Hong Kong People’s Republic of China

**Keywords:** Wearable supercapacitor, Conductive fiber, Computerized embroidering, Interdigital pattern, Cobalt phosphide

## Abstract

Wearable supercapacitors (SCs) are gaining prominence as portable energy storage devices. To develop high-performance wearable SCs, the significant relationship among material, structure, and performance inspired us with a delicate design of the highly wearable embroidered supercapacitors made from the conductive fibers composited. By rendering the conductive interdigitally patterned embroidery as both the current collector and skeleton for the SCs, the novel pseudocapacitive material cobalt phosphides were then successfully electrodeposited, forming the first flexible and wearable in-plane embroidery SCs. The electrochemical measurements manifested that the highest specific capacitance was nearly 156.6 mF cm^−2^ (65.72 F g^−1^) at the current density of 0.6 mA cm^−2^ (0.25 A g^−1^), with a high energy density of 0.013 mWh cm^−2^ (5.55 Wh kg^−1^) at a power density of 0.24 mW cm^−2^ (100 W kg^−1^). As a demonstration, a monogrammed pattern was ingeniously designed and embroidered on the laboratory gown as the wearable in-plane SCs, which showed both decent electrochemical performance and excellent flexibility.

**Electronic supplementary material:**

The online version of this article (10.1007/s40820-019-0321-x) contains supplementary material, which is available to authorized users.

## Highlights

- The conductive silver-plated nylon yarns fully fit the demand for wearable supercapacitor skeleton, owing to the advantages of high conductivity and flexibility.
- The computerized programming embroidering technique was firstly applied for realizing standardized batch processing of the flexible supercapacitor skeleton in various patterns.
- Cobalt phosphides were properly electrodeposited on the conductive embroidery as the pseudocapacitive materials, providing remarkable electrochemical performance.

**Electronic supplementary material:**

The online version of this article (10.1007/s40820-019-0321-x) contains supplementary material, which is available to authorized users.

## Graphical abstract

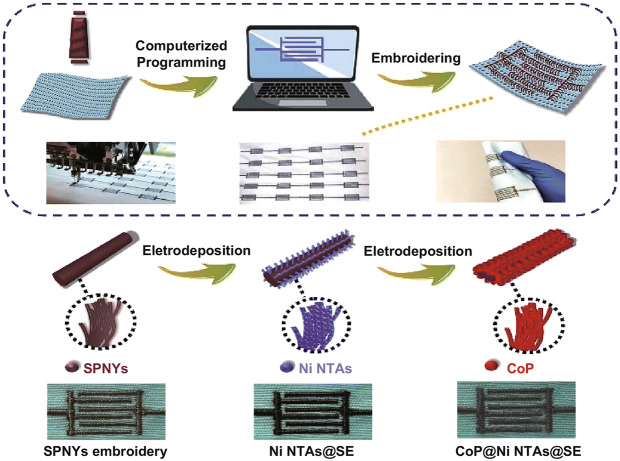

## Introduction

Wearable energy storage devices are receiving great attention and popularity for the growing demands from the modern portable electronics and smart textiles [1–4]. In particular, supercapacitors (SCs), also known as electrochemical capacitors, are drawing attention for their irreplaceable advantages in high power density and long cycle life, leading to a worldwide trend seeking for the novel and superior electroactive materials [5–8]. Among the hot-spot pseudocapacitive materials, the transition metal nickel- and cobalt-based compounds are widely studied owing to their high theoretical capacitance, such as Ni–Co oxides [9–11], Ni–Co hydroxides [12, 13], and Ni–Co sulfides [14–16]. However, for nickel and cobalt phosphides, more studies were carried out in the fields of hydrogen evolution and Na-ion batteries and interests casted on SCs are still far from enough from the perspective of electrochemical energy storage [17–20]. Reviewing the recent work of Ni/Co-related phosphides in SCs [21–23], Co_2_P nanoflowers- and graphene-based asymmetric SCs showed impressive electrochemical properties, with the energy density of 8.8 Wh kg^−1^ (at a high power density of 6 kWh kg^−1^) [24]. Also, a CoP nanowires-based carbon cloth displayed remarkable negative capacitive behavior in the neutral LiCl/PVA electrolyte, but it dissolved gradually due to the side reactions with Cl^−^ system [25]. In this regard, it is highly expected to further explore the potential of Ni/Co-based phosphides as the electrode material of SCs.

As a matter of fact, for flexible and wearable devices, the typical textile processing techniques [26–30], such as knitting, weaving, and embroidering, are rarely reported for integrating with the applications. Instead, more works paid their attention to the pure synthesis of the electroactive materials [31–33]. And the commonly reported flexible substrates mainly include carbon-derived materials (e.g., activated carbon (AC), carbon nanotubes (CNTs), and graphene) [34–36], polymer-based films (e.g., PET) [37–39], and metallic foils (e.g., Cu foil and Ti foil) [40, 41], which showed only limited flexibility when compared to the real garments. By contrast, the embroidery stitches, originating from the conventional hand sewing, can not only produce various patterns on the fabric, but also realize the standardized batch processing via the computer-aided programming techniques [42].

In this work, inspired by the design of the small in-plane SCs [43–45], a conductive silver-plated nylon yarns (SPNYs)-patterned embroidery was, for the first time, rationally designed and interdigitally constructed on the common fabric via the computerized programming. A thin layer of nickel nanothorn arrays (NTAs) was then electrodeposited on the conductive embroidery for restoring the silver surfaces and creating more porous active sites for loading CoP. Finally, the CoP microspheres were finely located on the Ni NTAs-covered embroidery skeleton, and the all-solid-state in-plane CoP@Ni NTAs@SE SCs were then assembled and measured, showing not only competitive capacitance but also extraordinary flexibility. Moreover, the computerized programming successfully embroidered a monogrammed conductive pattern on the laboratory gown, demonstrating a promising prospect in large-scale production of the wearable energy storage devices.

## Experimental

### Materials

Chemicals of LiOH·H_2_O 99%, NaH_2_PO_2_ 98%, CoCl_2_·6H_2_O 99%, NiCl_2_·6H_2_O 99%, NiSO_4_·6H_2_O 99%, CH_3_COONa 58%, H_3_BO_3_ 99%, Na_3_C_6_H_5_O_7_·2H_2_O 99%, polyvinyl alcohol (PVA), pyrrole 98%, H_2_SO_4_ 96%, HCl 37%, and absolute ethanol are all of analytical grade. Conductive silver-plated nylon yarns (20D, 15 Ω cm^−1^) were purchased from Qingdao Zhiyuanxiangyu Functional Fabric Co., Ltd.

### Fabrication of CoP-Decorated Patternable Conductive Embroidery SCs

#### Creation of the Conductive SPNYs Embroidery

TAJIMA wearable embroidery machine with the model of TCMX-600 was used for automatically making the conductive embroidery stitches with a stitching speed up to 750 rpm. The embroidery machine has a working space of D × W by 460 × 550 mm^2^, and the stitch length ranges from 0.1 to 12.1 mm. With the aid of computerized programming, various patterns were properly embroidered on the base fabrics. Instead of using the common cotton or polyester yarns, the highly conductive SPNYs were selected for creating the embroidery serving as both the substrates and current collectors for the in-plane SCs. The detailed pattern dimensions are shown in Fig. S1.

#### Pre-deposition of Ni Nanothorn on SPNYs Embroidery

Before the fabrication of the electroactive material CoP, a thin layer of nickel nanothorn arrays (NTAs) was preferentially deposited on the SPNYs. That is, a piece of the embroidered SE was immersed into a 50-mL beaker with 10% (volume concentration) H_2_SO_4_ solution for the surface pretreatment of 1 h. The fabric was then removed and washed with the deionized (DI) water, ethanol, and acetone in order, followed by a drying process in the vacuum oven for 2 h under 60 °C. The electrodeposition of the Ni NTAs was conducted via a chronoamperometry mode, with the pretreated SE as the working electrode, a stainless steel plate (3.0 × 4.0 cm^2^) as the counter electrode, and a saturated calomel electrode (SCE) as the reference electrode. The base electrolytic solution was composed of 30 mM NiSO_4_, 0.4 M H_3_BO_3_, and 0.2 M C_6_H_5_NA_3_O_7_·2H_2_O, with the pH around 4 ~ 5. To optimize the synthetic conditions, the electrodeposition was performed at various potentials (1.0, 1.2, 1.4, and 1.6 V), and the resulted products were then rinsed thoroughly by the DI water and stored in the vacuum oven under 60 °C.

#### Decoration of the CoP Microspheres on the Ni NTAs@SE

The cobalt phosphide was electrodeposited via the similar electrochemical configuration as Ni NTAs, while the electrolytic solution consisted of 10 mM CoCl_2_, 0.1 M Na_3_C_6_H_5_O_7_, 0.6 M NaH_2_PO_2_, with nitrogen bubbled through for 30 min before using. The electrodeposition was performed at different potentials (0.9, 1.1, and 1.3 V) and durations (500, 750, 1000, and 1250 s) for further confirming the most suitable conditions. The electrolytic solutions with different Ni/Co ratios (1:0, 1:1) were also applied for a comparison.

### Characterization and Electrochemical Measurements

The chemical phases of SE, Ni NTAs@SE, and CoP@Ni NTAs@SE were all determined by the X-ray diffraction (XRD: Cu target, Ka, *λ* = 0.15406 nm) with a Bruker D8 Advance X-ray diffractometer and a scanning transmission electron microscope (STEM: Jeol JEM-2100F). The morphologies were measured by an EDS detector equipped with scanning electron microscopy (SEM: TESCAN VEGA3).

Raman spectra were recorded via a micro-Raman spectroscope (JY-HR800) with the laser emitting at 532 nm. Fourier transform infrared spectroscopy (FTIR) was achieved by applying a Spectrum 100 spectrometer (PerkinElmer) in the range of 600–3500 cm^−1^ at a resolution of 4 cm^−1^.

The electrochemical measurements were performed at room temperature via an electrochemical workstation instrument of Princeton Versa STAT3. For the single-electrode measurement, a three-electrode electrochemical cell was applied, and a piece of platinum plate and a Hg/HgO electrode served as the counter and reference electrodes, respectively, and the as-prepared electrode acted as the working electrode, with a 1 M LiOH aqueous solution as the electrolyte. For the all-solid-state SC electrochemical measurement, one electrode of the in-plane SC acted as both the counter and reference electrodes, while another electrode served as the working electrode. The cyclic voltammetry (CV), galvanostatic charge and discharge (GCD), and the electrochemical impedance spectroscopy (EIS) were all applied. The gravimetric and areal capacitances derived from GCD curves were calculated by Eq. 1 [35, 46]:

1 $$C = I \cdot t/\left( {\Delta U \cdot m\left( S \right)} \right)$$

The energy density (*E*) and power density (*P*) of the device were all derived from Eqs. 2 and 3 [46]:

2 $$E = C \cdot \Delta U^{2} /\left( {2 \cdot 3600} \right)$$

3 $$P = 3600 \cdot E/\left( {\Delta t} \right)$$

where *I*, Δ*U*, Δ*t*, *m*, and *S* are the discharge current (A), the voltage window (V), the discharging duration (s), the mass loading of the electroactive CoP (g), and the surface area of the device (cm^2^). Specifically, the mass loadings of CoP were measured by the weighing samples before and after the CoP electrodeposition, and the interdigital fingers were approximated as elliptic cylinders for calculating the device surface area.

### Fabrication of All-Solid-State Embroidery SCs

The gel PVA/LiOH electrolyte was prepared by dissolving 5 g PVA and 1.2 g LiOH in 50 mL DI water at 85–90 °C for 1.5 h. The all-solid-state SCs were then assembled by pouring the as-prepared PVA/LiOH gel electrolyte on the electroactive materials-loaded embroidery and drying at room temperature for 2 h.

## Results and Discussion

### Characterization of CoP@Ni NTAs@SE

**Fig. 1:**
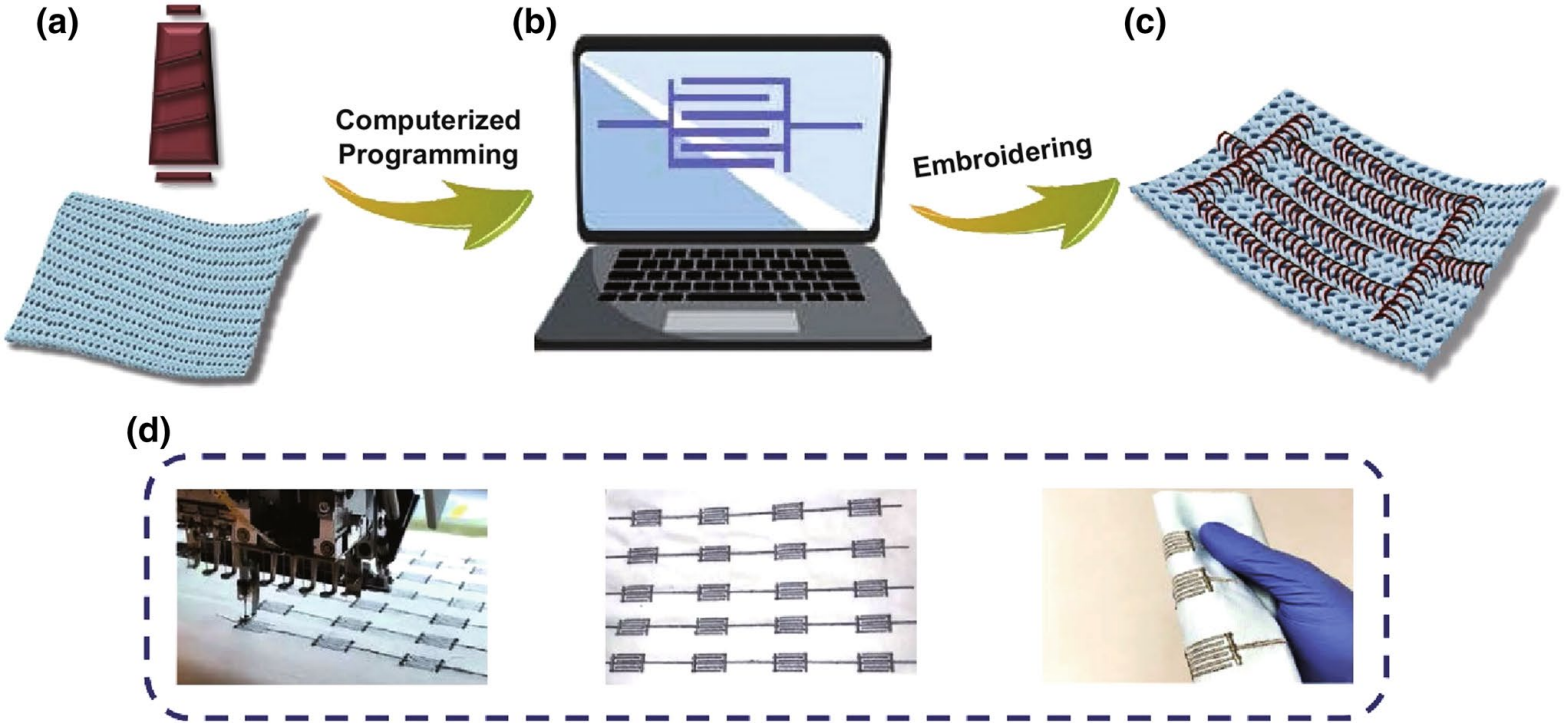
**a** Schematic diagram of a cone of the conductive silver-plated nylon yarns and a piece of the cotton fabric. **b** Diagram of the computer-aided programming. **c** Diagram of the conductive SE. **d** Real photographs of the SE products

The schematic diagram of the CoP@Ni NTAs@SE fabrication is demonstrated in Fig. 1a–c, where an interdigitally patterned embroidery was successfully developed via the computerized programming. Firstly, the target patterns were designed and drawn by the soft kit, and a piece of the SPNYs was then threaded into the embroidery machine for stitching the embroidery, with a piece of the common cotton fabric stuck on the workbench as the substrate. With the aid of the computerized wearable technology, the embroidering machine stitched the conductive SPNYs patterns on the cotton fabric automatically, and as shown in Fig. 1d, the conductive SE can be processed on a large scale. The resulted SE showed not only great conductivity, but also excellent flexibility. For a demonstration, the practical embroidering stitching was recorded as a short video file, as shown in the supporting files.

**Fig. 2:**
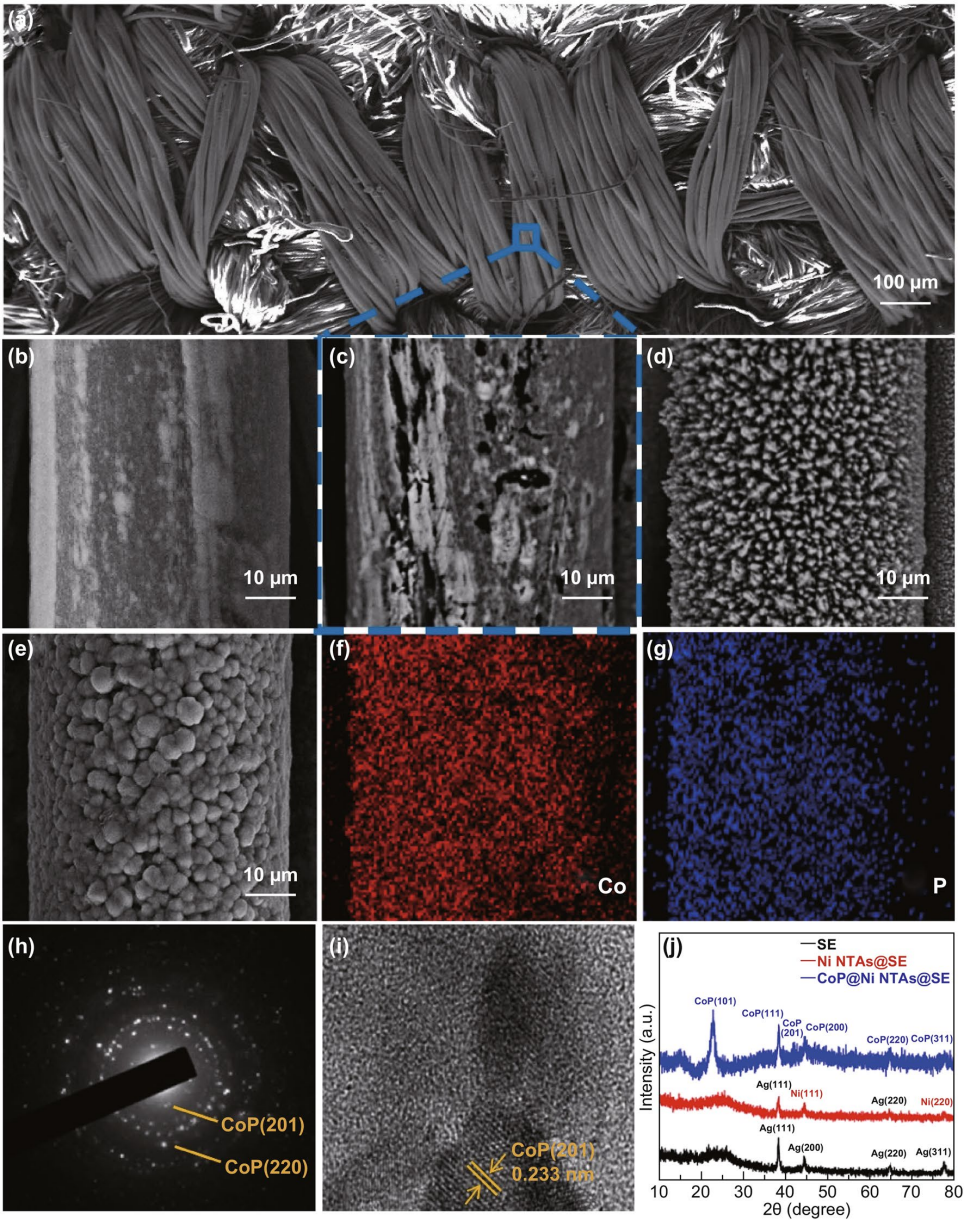
**a** SEM images of the overview of the SE single finger. **b** A single fiber of the original SPNYs. **c** Damaged fiber surface of the SPNYs. **d** A single fiber of the Ni NTAs. **e** Microspheres of the CoP@Ni NTAs@SE. **f–g** EDS mapping of Co and P. **h** SEAD pattern. **i** TEM image of CoP. **j** XRD curves of the SE, Ni NTAs@SE, and CoP@Ni NTAs@SE

**Fig. 3:**
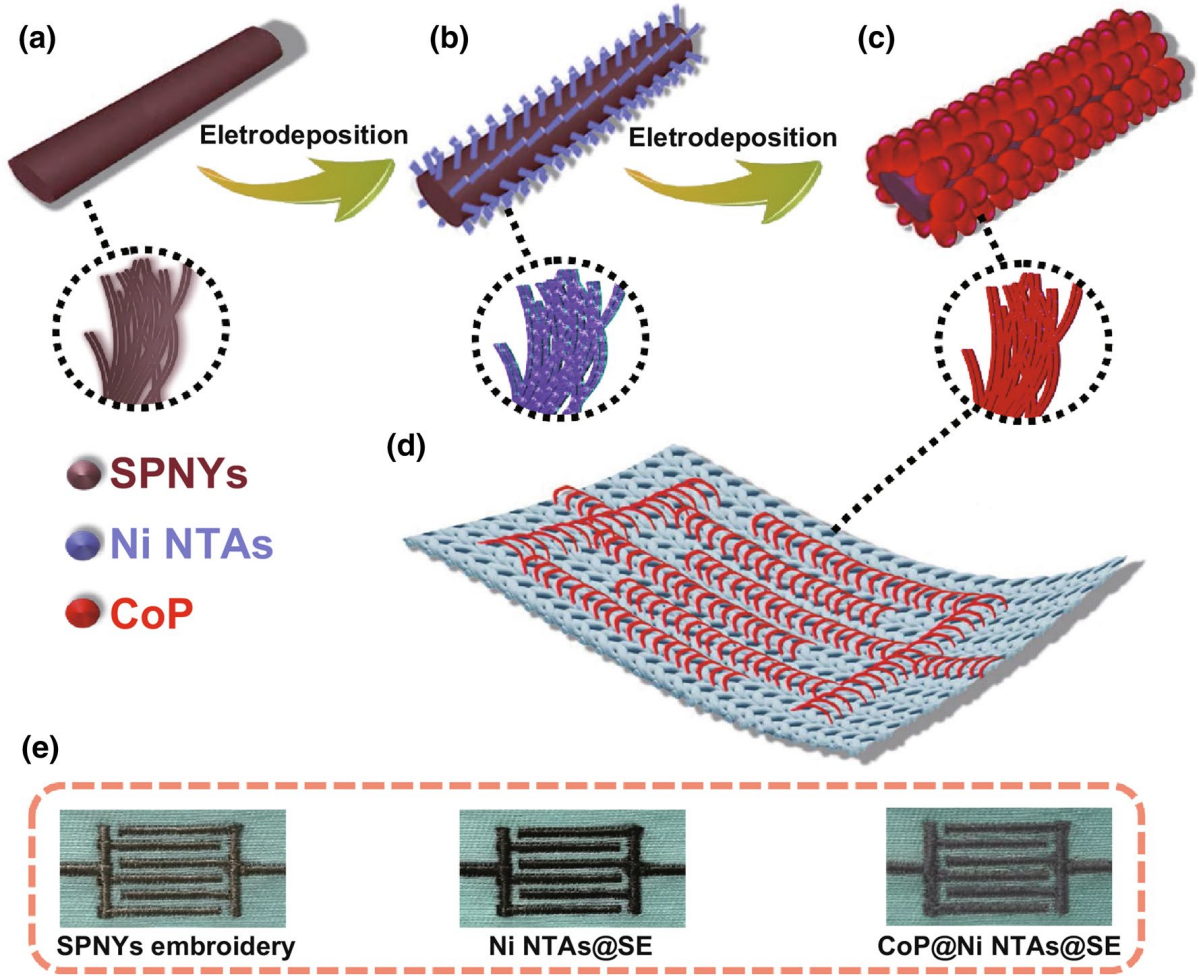
**a**–**c** Schematic diagram of the fabrication CoP@Ni NTAs@SE. **d** Overview of the CoP@Ni NTAs@SE. **e** Real images of the SE, Ni NTAs@SE and CoP@Ni NTAs@SE

**Fig. 4:**
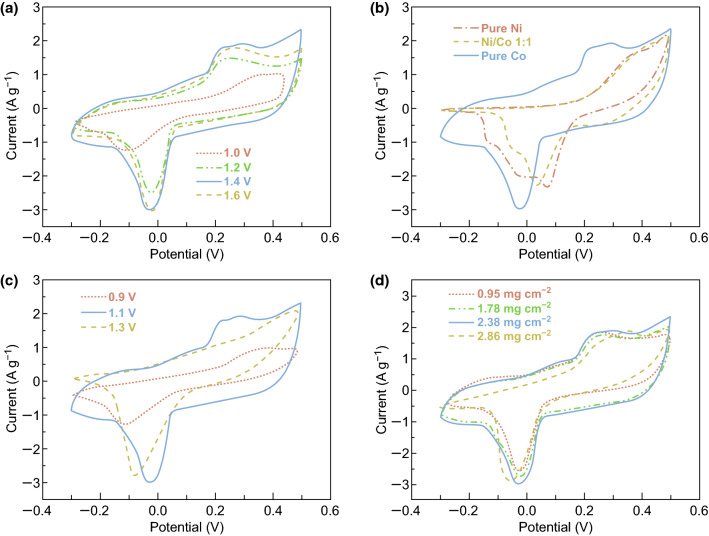
CV curves of **a** the electrodes with nickel layers electrodeposited at different voltages, **b** phosphides fabricated by different Ni/Co ratios, **c** CoP fabricated on Ni NTAs@SE at different voltages, **d** different CoP mass loadings on Ni NTAs@SE at a scanning voltage rate of 20 mV s^−1^

To synthesize the electroactive materials on the conductive fabric, electrodeposition is usually considered to be one of the effective approaches, owing to its significant advantages, like the rapid synthesis, the proper temperature, the high mass loadings, etc. [47, 48]. However, the conductivity of working electrode usually has a major impact on the electrodeposition [16], especially when the surface conductivity is inhomogeneous, and it would not deliver the expected results. Here, as compared to the SEM images of the original SPNYs in Fig. 2b, the microscopic surfaces of the embroidered SPNYs were slightly damaged, as shown in Fig. 2c, where the irregular defects clearly appeared on the SPNYs. Thus, to acquire a decent layer of the electroactive materials, the damaged surfaces of SE need to be further restored before the following steps. To restore the damaged SPNYs surfaces and provide homogeneously conductive substrate, a thin layer of the metallic nickel was created via the electrodeposition, as shown in Fig. 3a, b. For maximizing the role of the nickel layer, the electrodepositions with different voltages were conducted, respectively. As shown in Fig. S2a, very fine and dense nickel nanoparticles evenly wrapped the SPNYs when the electrodepositing voltages were lower than 1.2 V, and when the voltages were around 1.6 V, the nickel dendrites with lengths above tens of microns largely emerged, inevitably leading to the falling off of the materials, as shown in Fig. S2c. By contrast, nickel layer obtained at a moderate voltage of 1.4 V showed the very uniform morphology of nanorods or nanothorns (NTAs), as shown in Fig. 2d. To confirm the most advantageous nickel morphology for loading CoP, the cyclic voltammetry was then used to study the pseudocapacitive performances of CoP grown on the different nickel layers. Figure 4a shows that the gravimetric capacitance of CoP on nickel layer obtained at 1.4 V was higher than that on the smooth nickel surfaces (deposited lower than 1.2 V). And CoP on the nickel layer from 1.6 V exhibited disproportional capacitance degradations, due to the weak adhesion between the nickel dendrites and the SPNYs. Thus, 1.4 V was considered to be the most suitable voltage for the electrodeposition. To acquire a proper nickel thickness, the electrodepositing durations of 1000, 1500, 2000, and 2500 s were then performed, and it indicated that less and incomplete nickel layers were coated on SPNYs when the duration was too short, and large and longer Ni NTAs can be produced when the duration exceeds 2000 s. Therefore, the reasonable electrodepositing duration was confirmed to be 2000 s. The conductivities of SPNYs at different stages were tested by the four-point probe sheet resistance test, as listed in Table 1.

**Table 1:** Electrical resistances of different SPNYs

Yarn condition	Resistivity (Ω·cm·square^−1^)	Thickness (nm)
Pristine SPNYs	0.067	20–50
Embroidered SPNYs	0.635	20–50
Ni NTAs@SPNYs	0.294	800–1200
CoP@Ni NTAs@SPNYs	25.83	2000–3000

The electroactive material of CoP was then electrodeposited on the as-prepared Ni NTAs, as shown in Fig. 3c, d. Generally, studies on the nickel or cobalt composites are normally performed by investigating different Ni-to-Co ratios for gaining the optimal electrochemical performance [16, 49, 50]. In this regard, here, three distinct and empirical ratios (Ni/Co = 1:0, Ni/Co = 1:1, Ni/Co = 0:1) were carried out to make a comparison pending the capacitance between electrodes with different nickel-to-cobalt ratios. As shown in Fig. 4b, CV curves of both pure nickel phosphide and Ni/Co 1:1 phosphide showed much smaller loops than the pure cobalt phosphide, which means the mixing of nickel phosphide into the cobalt phosphide is almost in vain. Therefore, the electroactive material of the pure cobalt phosphide was used for the further study. Experiments with different CoP electrodepositing voltages and durations were conducted in order to gain the most suitable synthetic conditions. Figure 4c shows the CV curves of CoP formed at three different electrodepositing voltages (0.9, 1.1, and 1.3 V), where CoP obtained at 1.1 V displayed the highest electrochemical performance. Correspondingly, the SEM images of CoP obtained at 0.9 and 1.3 V are also shown in Fig. S3a, b for comparison. It can be seen that more dense and compact particles were formed at 0.9 V, and the microspheres covered by the irregular fakes or sheets (could be the Co(OH)_2_ formed by the hydrogen evolution [51, 52]) were obtained at 1.3 V, as shown in Fig. S3b. Notably, the unwanted impurities can negatively affect the CoP synthesis. By contrast, Fig. 2e shows that a layer of very uniform microspheres deposited on Ni NTAs@SE at a moderate voltage of 1.1 V, with a large number of macropores between each other, which could benefit the ion diffusion between the electrolyte and the electroactive materials. This is consistent with the above result from the CV curves, and it is reasonable to control the CoP electrodeposition at a moderate voltage of 1.1 V.

Subsequently, to investigate and control the optimum mass loading, Fig. 4d gives the information on CV curves based on CoP mass loadings at different electrodepositing durations, where the gravimetric capacitance enlarged gradually as the loading of CoP increased, from 0.95 mg cm^−2^ at 500 s to 2.38 mg cm^−2^ at 1000 s, but a degradation was observed at the mass loading of 2.86 mg cm^−2^ at 1250 s. Thus, it is better to control the CoP amount around 2.38 mg cm^−2^ obtained at the electrodepositing duration of 1000 s. Finally, the most suitable electrodepositing condition for fabricating the electrode is nickel layer 1.4 V at 2000 s and cobalt phosphide 1.1 V at 1000 s. The resulted electrode showed the weight percentages of silver-plated nylon yarns, Ni layer, and CoP layer were 67.63%, 21.97%, and 11.56%, respectively. The cross-section information of the resulted CoP@Ni NTAs@SE is also displayed in Fig. S5a, b, including the SEM image and real photograph, which shows that the interdigital fingers have the quasi-elliptical cross section, with the long and short axes around 1 and 0.7 mm.

EDS mapping images of the CoP microspheres are displayed in Fig. 2f, g, with the red and blue representing elements Co and P, respectively, and the spectrum in Fig. S4 clearly exhibits the existence of Co and P, with the atomic percentage ratio to be 53.28:46.72 (nearly 1:1). The SAED image in Fig. 2h also discloses a series of lattice planes, mainly including CoP (201) and CoP (220) from inside to outside. Figure 2i shows the high-resolution TEM image, and the lattice fringes have an interplane spacing of 0.233 nm corresponding to CoP (201) plane. The XRD curves in Fig. 2j show the strong peak situated at the 2θ of 38.18° in all three samples, representing the crystal plane of Ag (111). Interestingly, the peak strength became weaker in Ni NTAs@SE but stronger in CoP@Ni NTAs@SE, and this could be probably because of the coverage of nickel and CoP on the SPNYs, where the Ni NTAs decreased the silver exposure and the CoP reinforced the peak strength because of the crystal plane of CoP (111) in the same location. The strongest characteristic peak of CoP (101) was well located in 31.58°, and the other (200), (220), and (311) can be also properly observed, which is well in agreement with both the results of SEAD and XRD patterns. This further proved that CoP was successfully synthesized via the electrodeposition.

Raman spectra of SE in Fig. S6a showed two characteristic peaks at 1356 cm^−1^ (*D*-band) and 1590 cm^−1^ (*G*-band), indicating the structural defects and disorders of carbons in the nylon yarns. For the Ni NTAs@SE and CoP@Ni NTAs@SE, no more peaks were observed, and the *D*-band and *G*-band peaks were weakened disproportionally due to the encapsulation of Ni and CoP on the SPNYs, which is consistent with the previous studies [53, 54]. The FTIR result in Fig. S6b shows no significant difference among the three curves, and only weak peaks were observed in SE, which might be from the organic compositions in the thin silver particle-coated nylon yarns. This also laterally supports the aforementioned analysis.

### Electrochemical Properties of the All-Solid-State Embroidery Supercapacitor

**Fig. 5:**
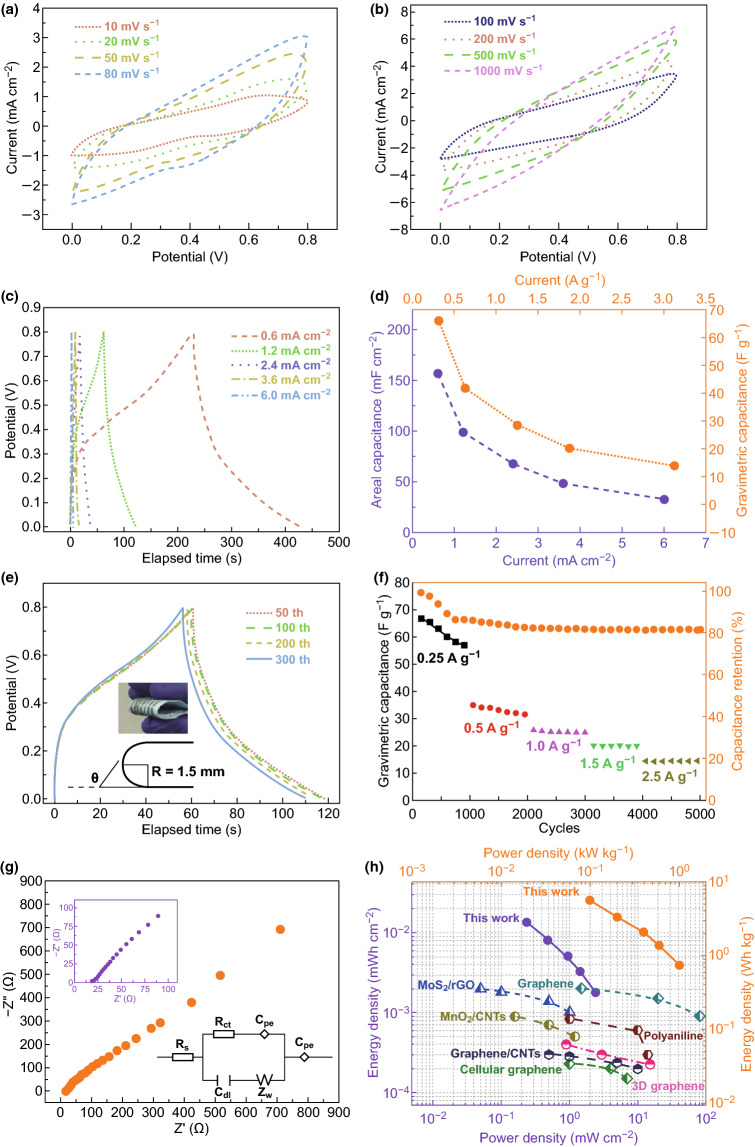
**a** CV curves of a CoP@Ni NTAs@SE-based SC at different low scanning voltage rates. **b** CV curves at different high scanning voltage rates. **c** GCD curves of a CoP@Ni NTAs@SE-based SC at different areal current densities. **d** Area and gravimetric capacitances at different current densities. **e** GCD curves of SC bent different times at 1.2 mA cm^−2^. **f** Cyclic stability within 5,000 times at different current densities. **g** Nyquist plots of the CoP@Ni NTAs@SE SC. **h** Ragone plot of the CoP@Ni NTAs@SE SC

An interdigitally patterned embroidery SC was successfully fabricated under the above conditions. As a matter of fact, patterns within the work limit of the embroidery machine are able to be knitted on the proper fabric substrate, and a demonstration is successfully shown in the following discussion. By pouring the gel electrolyte (1 M PVA/LiOH) on the CoP@Ni NTAs@SE, the electrochemical properties of one-piece all-solid-state SC were evaluated via the means of CV, GCD, and EIS. Figure 5a shows the CV curves of CoP@Ni NTAs@SE at the lower voltage scanning rate range, from 10 to 80 mV s^−1^. It can be seen that the typical pseudocapacitive redox peaks moved proportionally to the two ends of the voltage when the scanning rate increased, with the enclosed shape kept nearly original. The peak movement is mainly because of the polarization triggered by the fast electron migration and slow ion diffusion [34]. Similarly, higher voltage scanning rates were also applied, which unexpectedly showed the enlarged areas with almost the original shape, as shown in Fig. 5b. However, when the CV was carried out at the scanning rate of 1000 mV s^−1^, the corresponding curve was prone to the undesirable oval shape. Correspondingly, the GCD curves at different areal current densities were also displayed, as shown in Fig. 5c. It is noted that a voltage window of 0.8 V was acquired, which is closely matched with that of the CV curves. It also indicated that the voltage drop was about 0.04 V at the lowest current density of 0.6 mA cm^−2^ and increased to 0.32 V at the highest current density of 6 mA cm^−2^, displaying a low internal resistance [55, 56].

Based on the charge and discharge curves, both the areal and gravimetric capacitances were derived and calculated, as shown in Fig. 5d. The highest specific capacitance of 156.6 mF cm^−2^ (65.72 F g^−1^) was obtained at the current density of 0.6 mA cm^−2^ (0.25 A g^−1^). As the current density increased, both the areal and gravimetric capacitances dropped accordingly, and the lowest specific capacitance of 33.5 mF cm^−2^ (14.06 F g^−1^) was acquired at the highest current density of 6 mA cm^−2^ (2.5 A g^−1^). This is because the insufficient pseudocapacitive reactions occurred between the electrolytic ions and the electroactive materials at the very fast electrons embedding and disembedding processes. To examine the flexibility of the embroidery SC, the device was bent from 0° to 180° up to 300 times, with a bending radius of 1.5 mm. The corresponding curves to the 50th, 100th, 200th, and 300th GCD are displayed in Fig. 5e, respectively. It showed that within 200 times, the original curve shape was retained very well without significant changes, demonstrating a decent electrochemical stability under various deformations. However, the charging/discharging duration decreased greatly, when it cycled to the 300th time. This might be the result of structural damages from both the Ni layers and the CoP particles [57]. Figure 5f displays the cycling stability of the CoP@Ni NTAs@SE by charging and discharging up to 5000 times at various current densities. It was noted that the specific capacitance dropped more dramatically at the current density of 0.25 A g^−1^ in the first 1000 cycles, from 67.82 to 57.34 F g^−1^, and the corresponding capacitance retention declined to 86.11% at the same time. This is probably because the electrolyte ions diffuse adequately to the electroactive material surfaces at a low current density, where the redox was carried out more effectively than that at higher current densities, leading to more structural defects and much faster capacitance degradation [58, 59]. After increasing the charging and discharging currents, the capacitance was retained much better, with the capacitance loss less than 5% after the 5000th time.

To investigate the overall resistance, EIS was performed from 100 kHz to 0.01 Hz with a voltage amplitude of 5 mV as shown in Fig. 5g. As the inset displays, the intercept on the high-frequency region shows a low solution resistance, with about 18.42 Ω, reflecting the decent movement of the solid-state electrolyte ions. The moderate semicircle region represents the charge transfer resistance, which can be determined to be around 160 Ω. The linear part at the low-frequency region shows the typical Warburg impedance curve, with the phase angle greater than 45°. The moderate slope reflects that the diffusing condition within the electrode is one of the factors posing negative effects on the capacitive behavior of the device [60, 61]. The Ragone plot (energy density vs. power density) of the device is properly drawn for comparisons. As shown in Fig. 5h, the areal and gravimetric values are plotted in violet and orange, respectively, and it shows that a high energy density of 0.013 mWh cm^−2^ (5.55 Wh kg^−1^) was achieved at a power density of 0.24 mW cm^−2^ (100 W kg^−1^), which is comparable and even superior to the recently reported studies with the interdigital configuration based on MoS_2_@rGO (0.0019 mWh cm^−2^ at 0.08 mW cm^−2^) [38], 3D graphene (0.00042 mWh cm^−2^ at 1 mW cm^−2^) [62], polyaniline (0.00092 mWh cm^−2^ at 1 mW cm^−2^) [63], graphene film (0.0025 mWh cm^−2^ at 1.2 mW cm^−2^) [64], cellular graphene film (0.00022 mWh cm^−2^ at 0.98 mW cm^−2^) [65], graphene/CNTs film (0.00034 mWh cm^−2^ at 0.68 mW cm^−2^) [66], MnO_2_ CNTs (0.00088 mWh cm^−2^ at 0.16 mW cm^−2^) [67].

On the whole, the high performance of the CoP@Ni NTAs@SE SC can be summarized as follows: (1) The creative and advanced computerized embroidering techniques realized an interdigitally patterned conductive embroidery, (2) the conductive SPNYs-based embroidery substrate offered both fast electron transporting passage and excellent flexibility, (3) the subsequent Ni NTAs conductive layers provided not only improved conductivities, but also porous structures for loading the electroactive materials, and (4) the cobalt phosphides possess not only outstanding pseudocapacitance, but also a wide voltage window from negative to positive ranges.

### Application of the Embroidery SCs

**Fig. 6:**
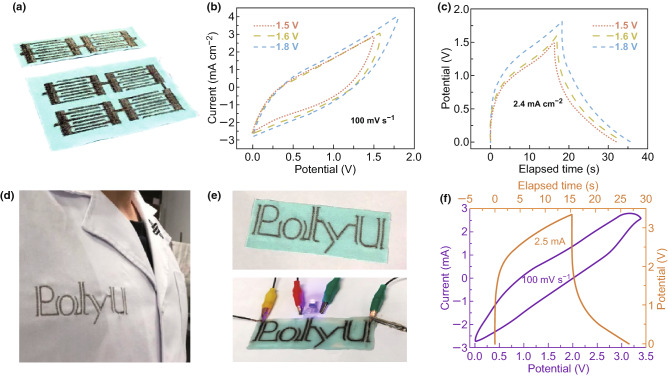
**a** Real photographs of two and four embroidery SCs connected in series. **b**, **c** CV and GCD curves of two SCs connected in series at a voltage scanning rate of 100 mV s^−1^ and current density of 2.4 mA cm^−2^. **d** Display of embroidering the “PolyU” monogrammed SC on the laboratory gown. **e** Electrochemical measurement on the “PolyU” SC. **f** CV and GCD curve of the “PolyU” SC

For a demonstration of the flexible and wearable SCs, various patterned embroideries were properly processed. As shown in Fig. 6a, two and four pairs of the embroidered SCs in series were successfully achieved. And the CV curves of two-pair devices are accordingly shown in Fig. 6b, where a wide range of the voltage windows from 1.5 to 1.8 V were explored and the curves acquired at the scanning rate of 100 mV s^−1^ almost kept unchanged. Similarly, the corresponding GCD curves are also plotted in Fig. 6c, and the as-obtained SCs were able to be charged and discharged within 1.8 V at the current density of 2.4 mA cm^−2^, yet with an aggravated IR drop, possibly caused by the extended series circuit [68]. Figure 6d displays the embroidered SCs on the real garment, where the “PolyU” monogrammed SCs were suitably created. A short video is attached in the supporting file (Video S2) for displaying the practical embroidering process. Besides, the corresponding CV and GCD curves are also plotted in Fig. 6f, and the pattern-embroidered SCs successfully powered a LED in Fig. 6e. But, it was also noted that the CV loop is not as symmetric as that of SCs with interdigital configuration, and this is mainly caused by the increase in the series resistance arising from the complicated “PolyU” monogrammed circuit.

## Conclusions

By implementing the strategy of loading novel pseudocapacitive material on the flexible textiles, the embroidered in-plane supercapacitors were successfully created via the computer-aided textile technology and the electrochemical synthesis approaches. Great flexibility and remarkable electrochemical performances were accordingly achieved owing to the porous nickel nanothorns-anchored CoP microspheres. Finally, a monogrammed SC was properly embroidered in series on the laboratory gown, inspiring a promising perspective for integrating more wearable technologies into the next generation of wearable energy storage devices.

## Electronic supplementary material

Below is the link to the electronic supplementary material.

Supplementary material 1 (PDF 261 kb)

Supplementary material 2 (MP4 1544 kb)

Supplementary material 3 (MP4 7707 kb)
