# Supplementary material for: Toward Flexible and Wearable Embroidered Supercapacitors from Cobalt Phosphides-Decorated Conductive Fibers
Source: Nanomicro Lett. 2019 Oct 17;11:89. doi: 10.1007/s40820-019-0321-x (PMC7770848; doi:10.1007/s40820-019-0321-x)
Supplement: Supplementary file 1 — Supplementary material 1 (PDF 261 kb) [file 40820_2019_321_MOESM1_ESM.pdf]

Supporting Information for

Towards Flexible and Wearable Embroidered Supercapacitors from Cobalt Phosphides Decorated Conductive Fibers

Jianfeng Wen[1], Bingang Xu[1,*], Jinyun Zhou[1]

[1]Nanotechnology Center, Institute of Textiles and Clothing, the Hong Kong Polytechnic University, Hung Hom, Kowloon, Hong Kong, China

*Corresponding author. E-mail: tcxubg@polyu.edu.hk (Bingang Xu)

S1 The Interdigital Patterns

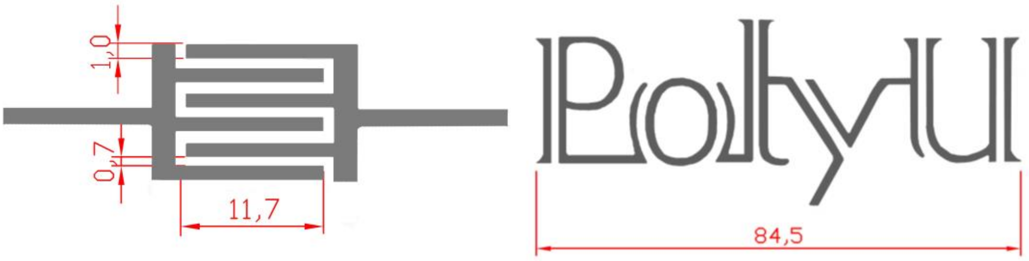

Fig. S1 The predesigned specifications and dimensions of the in-plane SCs in mm

S2 SEM Images of Nickel Layers at Different Electrodepositing Voltages

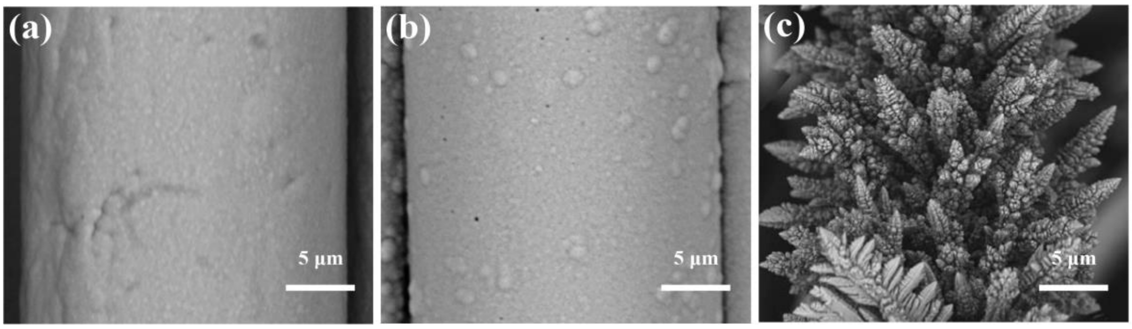

Fig. S2 SEM images of smooth nickel layer at **a** 1 V, **b** 1.2 V, **c** dendrite nickel layer at 1.6 V

S3 SEM Images of CoP@Ni NTAs@SE at Different Electrodepositing Voltages

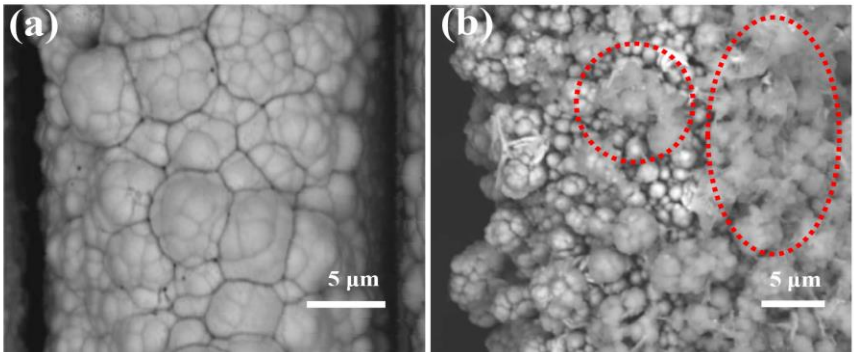

Fig. S3 SEM images of CoP obtained at **a** 0.9 V, **b** 1.3 V

S4 Images of CoP@Ni NTAs@SE Cross Section

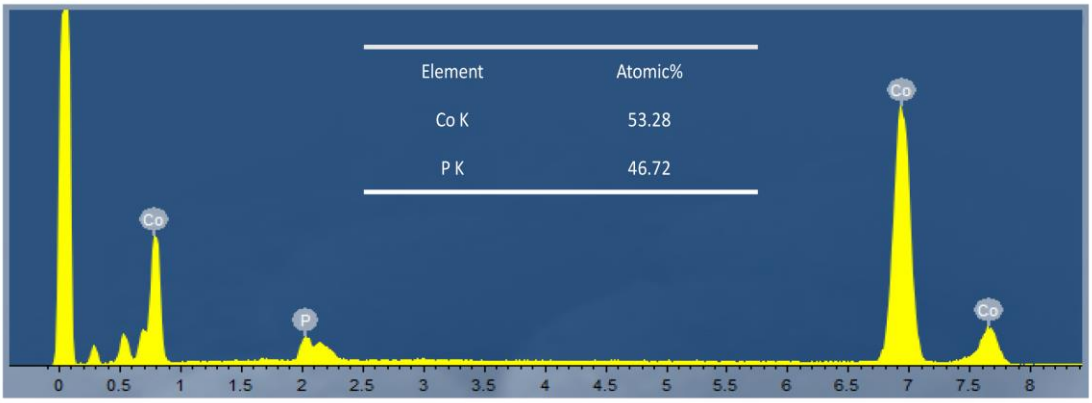

Fig. S4 The EDS spectrum of CoP@Ni NTAs@SE

S5 Images of CoP@Ni NTAs@SE Cross Section

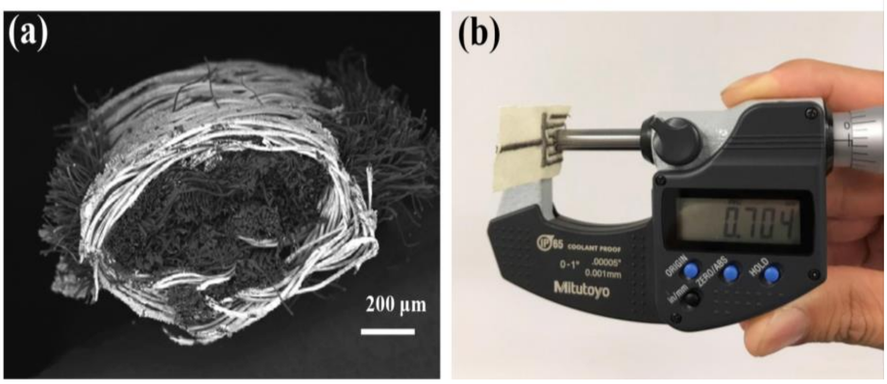

Fig. S5 a SEM cross section image, **b** Real photo of CoP@Ni NTAs@SE

S6 Characterization of Raman and FTIR

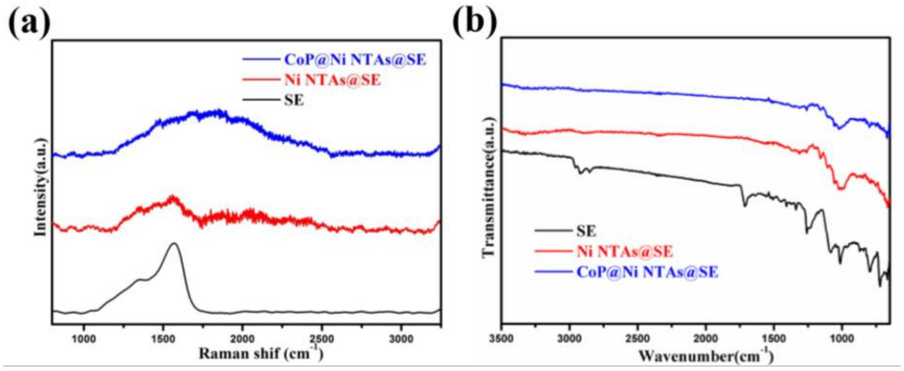

Fig. S6 a Raman spectrums and **b** FTIR spectrums of SE, Ni NTAs@SE, and CoP@Ni NTAs@SE
